# Long-term air pollution exposure and cardiovascular disease risk across cardiovascular-renal-metabolic stages: a nationwide study

**DOI:** 10.1186/s12889-025-23348-1

**Published:** 2025-07-02

**Authors:** Leying Zhao, Cong Zhao, Weiwei Sun, Huijuan Zheng, Yabin Gao, Chan Kam Wa, Qiong Wang, Qingqing Liu, Yaoxian Wang, Zhen Wang

**Affiliations:** 1 Dongzhimen Hospital, Beijing University of Chinese Medicine, Beijing, 100700 China; 2 Beijing University of Chinese Medicine, Beijing, China; 3 Department of Nephropathy, The First Affiliated Hospital of Henan University of Chinese Medicine, Zhengzhou, China; 4 School of Chinese Medicine, Hong Kong Baptist University, Hong Kong, China; 5 Vincent V.C. Woo Chinese Medicine Clinical Research Institute, Hong Kong Baptist University, Hong Kong, China; 6 CDC Key Laboratory of Environment and Population Health, National Institute of Environmental Health, Chinese Center for Disease Control and Prevention, Beijing, China; 7 Renal Research Institution of Beijing University of Chinese Medicine, Beijing, 100029 China; 8 Henan University of Chinese Medicine, Zhengzhou, 450046 China

**Keywords:** Air pollution, Cardiovascular disease, Cardiovascular-renal-metabolic syndrome, Long-term exposure

## Abstract

**Background:**

Cardiovascular-renal-metabolic (CKM) syndrome substantially elevates the risk of cardiovascular disease (CVD). Environmental air pollution, especially particulate matter (PM), is a key contributor, yet its long-term effects across CKM stages remain unclear.

**Objectives:**

This study aimed to evaluate the association between long-term exposure to different sizes of particulate matter (PM_1_, PM_2.5_, PM_10_) and CVD risk across the four stages of CKM syndrome.

**Methods:**

We conducted a nationwide prospective cohort study using data from the China Health and Retirement Longitudinal Study (CHARLS, 2011–2018), including 5,824 participants aged 45 years or older. CKM stages (0 to 3) were classified according to American Heart Association guidelines. Annual average concentrations of PM_1_, PM_2.5_, and PM_10_ were used to estimate individual exposure. Cox proportional hazards models were used to calculate adjusted hazard ratios (HRs), and population attributable fractions (PAFs) were estimated to assess the burden of air pollution on CVD.

**Results:**

During a median follow-up of 7 years, participants in the highest exposure group of PM_2.5_ had significantly increased CVD risk (HR = 2.31, 95% CI: 2.00–2.66). The risk rose progressively with CKM stage, peaking in stage 3 for PM_1_ (HR = 3.32, 95% CI: 2.24–4.92). PM_1_ and PM_2.5_ showed nonlinear exposure–response patterns, with sharply increasing CVD risk at higher concentrations. The highest PAF (~ 38%) occurred in CKM stage 2 under high PM_2.5_ exposure, indicating substantial burden among intermediate and advanced CKM stages. Among patients with chronic kidney disease, the association was attenuated, potentially due to medication use.

**Conclusions:**

Long-term exposure to ambient particulate matter significantly increases CVD risk, especially among individuals in advanced CKM stages.

**Implications:**

These findings support incorporating CKM staging into environmental health risk assessments and highlight the need for targeted cardiovascular screening and pollution control strategies in high-exposure regions.

**Clinical trial number:**

Not applicable.

**Supplementary Information:**

The online version contains supplementary material available at 10.1186/s12889-025-23348-1.

## Introduction

Cardiovascular diseases (CVD) represent the leading cause of global disease burden. According to the Global Burden of Disease Study 2019, East Asia experienced the most significant increases in both the incidence and mortality rates of CVD, surpassing all other regions [1]. Environmental air pollution is a significant contributor to CVD, with nearly 20% of CVD-related deaths attributable to air pollution [2, 3]. Among air pollutants, fine particulate matter (PM_2.5_) is identified as a primary risk factor for CVD due to its ability to deeply penetrate the lungs and induce systemic inflammation [4]. Ultrafine particles (PM_1_), due to their higher specific surface area and enhanced penetration capability, have also gained attention in recent years for their potential hazards [5].

The American Heart Association (AHA) has proposed a structured staging system (stages 0 to 3) to characterize the progressive severity of cardiovascular-renal-metabolic (CKM) syndrome, which encompasses obesity, insulin resistance, and coexisting dysfunctions of the cardiovascular, renal, and metabolic systems [6]. While air pollution is a recognized driver of cardiovascular morbidity, few epidemiological studies have investigated whether its impact on CVD risk varies by CKM stage. Understanding this potential interaction is essential to identify the most vulnerable subgroups and to inform precision environmental health interventions [7, 8].

Although previous studies have established strong associations between air pollution and individual components of CKM—such as chronic kidney disease, type 2 diabetes, and hypertension [9–11]—there remains a lack of comprehensive assessments that consider CKM as an integrated, stage-based construct [12]. Furthermore, to our knowledge, no nationwide longitudinal cohort has systematically examined how long-term exposure to particulate matter influences CVD risk across the full spectrum of CKM stages. This knowledge gap hampers the development of risk-stratified environmental health strategies and precision prevention for vulnerable cardiometabolic subgroups.

To address this gap, the present study aimed to evaluate the association between long-term exposure to different sizes of particulate matter (PM_1_, PM_2.5_, and PM_10_) and the risk of incident CVD in a large, nationally representative cohort of middle-aged and older adults in China. We further sought to assess whether this association is modified by the severity of CKM syndrome. We hypothesized that individuals at more advanced CKM stages would exhibit stronger associations between particulate matter exposure and CVD risk.

## Methods

### Data sources and study population

This study was a prospective, population-based cohort analysis using nationally representative data from the China Health and Retirement Longitudinal Study (CHARLS), a longitudinal survey targeting Chinese adults aged 45 and older. CHARLS began in 2011 and conducted regular surveys through 2018. Participants were selected using a multistage, stratified, probability-proportional-to-size (PPS) sampling strategy, covering both urban and rural areas across 28 provinces and 150 counties (districts) in China. To enhance national representativeness, post-stratification weights were calculated based on the 2010 Chinese Census. The design and cohort characteristics of CHARLS have been extensively documented in prior literature. All interviews were conducted using standardized CHARLS questionnaires, which were adapted from internationally validated aging studies. Field staff underwent systematic training and conducted face-to-face interviews using Computer-Assisted Personal Interviewing (CAPI) systems, with built-in validation checks, GPS tracking, photo verification, and audio recordings to ensure data integrity [13]. Physiological and biochemical measurements—including blood pressure and blood samples—were obtained using standardized clinical procedures. CHARLS adheres to the principles of the Declaration of Helsinki and has received approval from the Institutional Review Board (IRB) of Peking University (IRB 00001052–11015). All participants provided written informed consent before their involvement in the study.

Participants interviewed during the 2011–2012 wave were considered the baseline cohort and followed through 2018. Individuals aged ≥ 45 years with complete baseline demographic and clinical data, as well as valid follow-up information, were eligible for inclusion. Participants with pre-existing cardiovascular disease at baseline or missing key exposure or outcome information were excluded. While potential selection bias due to attrition or non-response may exist, the original sampling design and weighting strategy of CHARLS reduce systematic bias and enhance generalizability. The detailed inclusion and exclusion process is shown in the flowchart (Additional file 1: Fig. S1).

### Measurement of environmental air pollution exposure

In this study, we utilized annual 1 km high-resolution data on PM_1_, PM_2.5_, and PM_10_ from the China High Air Pollutants (CHAP) dataset (https://weijing-rs.github.io/product.html). The CHAP dataset is a high-quality air pollution dataset that has been widely used in related research. It provides the geographical distribution of various pollutants at high resolution, ensuring accurate exposure estimates [14–17]. To reflect long-term exposure to air pollution, we calculated the average pollutant concentration from the baseline survey to the final follow-up. Each participant’s air pollution exposure was matched to the county-level geographic coding corresponding to their residential address.

### Definition of stages 0–3 of CKM syndrome

The stages of CKM syndrome, ranging from 0 to 3, are classified according to the American Heart Association (AHA) Presidential Advisory Statement on CKM Syndrome [18]. Stage 0: No risk factors for CKM syndrome present. Stage 1: Characterized by excessive or dysfunctional adiposity. Stage 2: Involves the presence of metabolic risk factors and/or chronic kidney disease (CKD). Stage 3: Encompasses subclinical cardiovascular disease. In this study, we defined subclinical cardiovascular disease as individuals with a 10-year high CVD risk, calculated using the China-PAR project’s Chinese Atherosclerotic Cardiovascular Disease (ASCVD) risk prediction equation, or those with very high-risk CKD (stages G4 or G5) [19]. We estimated the glomerular filtration rate (eGFR) using the Chinese Modification of Diet in Renal Disease (C-MDRD) formula and classified participants into CKD stages according to the Kidney Disease: Improving Global Outcomes (KDIGO) guidelines [18, 20].

### Outcome assessment

The primary endpoint of this study was the incidence of CVD during the follow-up period from 2013 to 2018. Consistent with previous studies, information on self-reported physician-diagnosed CVD was collected using standardized questions: “Have you been diagnosed with [heart attack, coronary heart disease, angina, congestive heart failure, or other heart problems] by a doctor?” [21]. The CHARLS research team implemented stringent data recording and validation measures to ensure the reliability of the data [13].

### Covariates

In this study, covariate selection was based on potential confounding factors that could be associated with both air pollution exposure and the risk of CVD. These covariates include sociodemographic characteristics, lifestyle factors, health-related variables, and indoor air pollution [22]. Sociodemographic information was collected by trained interviewers and included sex, age, ethnicity, and education level. Lifestyle factors assessed included smoking and alcohol consumption. Health-related variables were obtained through blood tests and questionnaires and included Total Cholesterol (TC), High-Density Lipoprotein Cholesterol (HDL-C), Platelet Count (PLT), Blood Urea Nitrogen (BUN), C-Reactive Protein (CRP), and Uric Acid (UA). The presence of pulmonary diseases was determined based on participants’ self-reported physician diagnoses. Depressive symptoms were assessed using the 10-item Center for Epidemiologic Studies Depression Scale (CES-D-10) [23], a widely used instrument that has been validated in Chinese older adult populations [23]. A total score of 10 or above was used to define elevated depressive symptoms, consistent with established cut-off values in previous studies. Indoor air pollution was assessed by the type of cooking fuel used (solid fuel or clean fuel). All covariates were collected at baseline to ensure they could account for potential confounding effects during the study period.

### Data analysis

Participants were categorized into three groups based on exposure levels to different air pollutants: Q1 for low-level exposure, Q2 for medium-level exposure, and Q3 for high-level exposure. Descriptive statistics were used to analyze baseline characteristics across the entire cohort and among different CKM stages. No a priori sample size calculation was conducted as this study was a secondary analysis of an existing cohort. However, most exposure and outcome strata had sufficient events and participants (generally > 300), allowing robust estimation. We conducted univariate and multivariate Cox regression analyses to prospectively examine the relationship between various air pollutants and CVD incidence. We evaluated the proportional hazards assumption for all Cox models using Schoenfeld residuals, confirming that it was not violated. Variance inflation factors were calculated to assess multicollinearity among covariates, and no meaningful collinearity was identified. Selection of covariates was guided by existing literature and informed by biological plausibility [24]. Stratified analyses were conducted to assess the impact of CKM stages 0–3 and different levels of air pollutant exposure (low, medium, high) on CVD incidence. To address potential confounding, we adopted a stepwise model adjustment strategy based on literature and biological plausibility. Model 1: Adjusted for sociodemographic factors, including sex, age, residence, education level, marital status, smoking history, and alcohol consumption. Model 2: Further adjusted for health-related variables, pulmonary disease history, and depressive symptoms. Model 3: Additionally adjusted for indoor air pollution, assessed by the use of solid fuel for cooking. To further explore the concentration-response relationship between different air pollutants and CVD incidence, we employed restricted cubic splines (RCS) with three knots at the 10th, 50th, and 90th percentiles. We calculated the population attributable fraction (PAF) for different levels of air pollution exposure in CKM stages 0–3 using the Miettinen equation: $\:PAF=1-\frac{R{R}_{0}}{{\int\:}_{x=l}^{u}R{R}_{x}{p}_{x}dx}$ [25]. Additionally, subgroup analyses were performed by stratifying the study population based on sex, age, education level, marital status, residence, CKM stage, presence of diabetes, metabolic syndrome, and chronic kidney disease, to examine potential interaction effects.

To assess the robustness of our findings, we performed sensitivity analyses using data from 2015 as the baseline and CVD incidence from 2018 as the outcome. Univariate and multivariate Cox regression models were applied to evaluate the relationship between different air pollutants and CVD incidence, and subgroup analyses were performed to observe potential interaction effects between air pollution and CKM stages. All statistical analyses were conducted using R Studio software (version 4.4.1), with a two-sided *P*-value of less than 0.05 considered statistically significant.

## Results

### Baseline characteristics

A total of 17,705 participants were originally enrolled in the 2011–2012 baseline wave of the CHARLS cohort. After applying the exclusion criteria, we obtained a final analytic sample of 5,824 individuals. Specifically, we excluded: 2,440 participants with self-reported physician-diagnosed cardiovascular disease at baseline; 4,666 participants due to missing information on CVD history before outcome events, lack of valid address for follow-up, or relocation during the follow-up period; 4,775 participants lacking key diagnostic information required for CKM staging.

Table 1 presents the baseline characteristics of the CHARLS participants. Among the 5,824 participants, the mean age was 58.14 ± 9.05 years, with females representing 54.81% and males 45.19%. The distribution across CKM stages was as follows: 657 participants in stage 0, 1,260 in stage 1, 2,711 in stage 2, and 1,196 in stage 3. Adults in CKM stage 3 were more likely to be older, male, have lower education levels, and be unmarried. Smoking and drinking habits were more prevalent in the CKM stage 3 group. Levels of BUN, FBG, and Scr were significantly higher in CKM stage 3 compared to other stages, while eGFR was the lowest. Conversely, levels of TC and CRP were lowest in stage 0. The prevalence of pulmonary diseases and depression did not differ across CKM stages, but the prevalence of hypertension and diabetes increased with higher CKM stages. The average exposure levels for PM_1_, PM_2.5_, and PM_10_ were 32.62 ± 9.91 µg/m^3^, 53.20 ± 17.15 µg/m^3^, and 91.06 ± 31.63 µg/m^3^, respectively.

**Table 1:** Baseline characteristics of the enrolled participants according to CKM stages

	Total (*n* = 5824)	0 stage (*n* = 657)	1 stage (*n* = 1260)	2 stage (*n* = 2711)	3 stage (*n* = 1196)	*P* value
Age, years	58.14 ± 9.05	56.46 ± 8.20	55.14 ± 7.61	56.52 ± 8.14	65.90 ± 8.61	< 0.0001
Sex						< 0.0001
Female	3192(54.81)	286(43.53)	740(58.73)	1904(70.23)	262(21.91)	
Male	2632(45.19)	371(56.47)	520(41.27)	807(29.77)	934(78.09)	
Marital						< 0.0001
Married	5247(90.09)	603(91.78)	1157(91.83)	2472(91.18)	1015(84.87)	
Not married	577(9.91)	54(8.22)	103(8.17)	239(8.82)	181(15.13)	
Education						< 0.0001
Above high school	466(8.00)	65(9.89)	108(8.57)	236(8.71)	57(4.77)	
Below high school	5357(92.00)	592(90.11)	1152(91.43)	2475(91.29)	1138(95.23)	
Waist Circumference, cm	84.08 ± 12.11	74.45 ± 8.61	82.61 ± 10.22	86.01 ± 11.60	86.55 ± 13.80	< 0.0001
Body Mass Index, kg/m^2^	23.49 ± 3.80	20.17 ± 1.68	23.13 ± 3.15	24.35 ± 3.96	23.74 ± 3.86	< 0.0001
Smoking Status						< 0.0001
Current	1740(29.88)	277(42.16)	334(26.51)	533(19.66)	596(49.83)	
Former	457(7.85)	38(5.78)	77(6.11)	176(6.49)	166(13.88)	
Never	3627(62.28)	342(52.05)	849(67.38)	2002(73.85)	434(36.29)	
Drinking Status						< 0.0001
Current	789(13.55)	81(12.33)	151(11.98)	302(11.14)	255(21.32)	
Former	1622(27.85)	233(35.46)	351(27.86)	625(23.05)	413(34.53)	
Never	3413(58.60)	343(52.21)	758(60.16)	1784(65.81)	528(44.15)	
PLT, ×10^9^/L	212.67 ± 77.63	210.23 ± 74.37	211.69 ± 86.94	213.69 ± 73.39	212.71 ± 78.34	0.40
BUN, mg/dL	15.63 ± 4.43	15.40 ± 4.24	15.55 ± 4.26	15.35 ± 4.33	16.48 ± 4.82	< 0.0001
FBG, mg/dL	108.68 ± 33.54	91.01 ± 7.88	100.67 ± 10.17	111.09 ± 32.59	121.37 ± 50.17	< 0.0001
Scr, mg/dL	0.77 ± 0.18	0.75 ± 0.15	0.72 ± 0.14	0.76 ± 0.18	0.85 ± 0.21	< 0.0001
TC, mg/dL	194.17 ± 38.45	179.96 ± 32.26	188.35 ± 34.88	198.72 ± 39.35	197.79 ± 40.40	< 0.0001
TG, mg/dL	128.97 ± 104.18	77.82 ± 25.14	82.19 ± 24.70	153.67 ± 105.92	150.39 ± 144.44	< 0.0001
HDL - C, mg/dL	51.72 ± 15.20	58.68 ± 14.85	57.93 ± 13.76	49.22 ± 14.78	47.04 ± 14.40	< 0.0001
LDL - C, mg/dL	117.35 ± 34.74	108.07 ± 29.46	116.17 ± 30.77	118.59 ± 35.96	120.87 ± 37.59	< 0.0001
CRP, mg/dL	2.48 ± 6.92	2.22 ± 6.36	2.35 ± 7.99	2.34 ± 6.49	3.07 ± 6.91	0.01
HbA1c, %	5.26 ± 0.78	5.00 ± 0.35	5.12 ± 0.38	5.30 ± 0.82	5.47 ± 1.07	< 0.0001
eGFR, ml/min/1.73m^2^	123.22 ± 30.62	130.06 ± 26.65	129.48 ± 26.70	120.93 ± 32.30	118.05 ± 30.90	< 0.0001
Lung Disease	528(9.09)	64(9.76)	101(8.05)	241(8.91)	122(10.23)	0.27
Depression	2015(35.46)	245(37.87)	416(33.85)	964(36.43)	390(33.62)	0.12
Hypertension	2139(36.73)			1294(47.73)	845(70.65)	< 0.0001
Diabetes	272(4.72)			148(5.51)	124(10.51)	< 0.0001
Metabolic Syndrome	2345(40.26)			1692(62.41)	653(54.60)	< 0.0001
Ambient Air Pollutants						
PM_1_, µg/m^3^	32.62 ± 9.91	29.97 ± 9.31	32.98 ± 9.44	32.53 ± 9.97	33.90 ± 10.28	< 0.0001
PM_10_, µg/m^3^	91.06 ± 31.63	82.13 ± 29.21	90.66 ± 30.65	90.58 ± 32.12	97.50 ± 31.46	< 0.0001
PM_25_, µg/m^3^	53.20 ± 17.15	48.19 ± 16.02	53.42 ± 16.45	53.01 ± 17.14	56.14 ± 17.82	< 0.0001

### Association between air pollution exposure and CVD incidence

In this study, with a median follow-up period of 7 years, high exposure to PM_1_, PM_2.5_, and PM_10_ was significantly associated with increased CVD incidence (Table 2). To assess the relationship between air pollution and CVD risk across CKM stages 0–3, three Cox proportional hazards models were constructed. In the unadjusted model, the high exposure group for PM_2.5_ exhibited a significantly increased CVD risk (HR = 2.18, 95% CI: 1.90–2.50, *P* < 0.0001). After adjusting for sociodemographic variables and lifestyle factors in Model 1, the CVD risk in the high exposure group remained elevated (HR = 2.20, 95% CI: 1.92–2.53, *P* < 0.0001). Further adjustment for physiological indicators in Model 2 did not substantially alter the risk estimate (HR = 2.31, 95% CI: 2.00–2.67, *P* < 0.0001). In Model 3, which additionally adjusted for indoor air pollution, the risk remained significant (HR = 2.31, 95% CI: 2.00–2.66, *P* < 0.0001). This pattern of association was consistent for PM_1_ and PM_10_ exposure.

**Table 2:** Multivariable Cox regression results for air pollutant exposure and incident CVD

	PM_1_				PM_2.5_				PM_10_			
	Q2	*P*	Q3	*P*	Q2	*P*	Q3	*P*	Q2	*P*	Q3	*P*
crude model	1.13(0.98,1.30)	0.10	1.52(1.33,1.74)	< 0.0001	1.12(0.96,1.31)	0.14	2.18(1.90,2.50)	< 0.0001	1.14(0.97,1.33)	0.10	2.23(1.94,2.57)	< 0.0001
Model 1	1.1(0.95,1.27)	0.20	1.52(1.32,1.74)	< 0.0001	1.12(0.96,1.31)	0.15	2.2(1.92,2.53)	< 0.0001	1.15(0.98,1.35)	0.08	2.29(1.99,2.64)	< 0.0001
Model 2	1.16(1.00,1.35)	0.05	1.55(1.35,1.79)	< 0.0001	1.2(1.02,1.41)	0.03	2.31(2.00,2.67)	< 0.0001	1.23(1.04,1.45)	0.01	2.45(2.11,2.85)	< 0.0001
Model 3	1.16(1.00,1.35)	0.05	1.55(1.34,1.78)	< 0.0001	1.19(1.02,1.40)	0.03	2.31(2.00,2.66)	< 0.0001	1.23(1.04,1.45)	0.01	2.45(2.11,2.84)	< 0.0001

Low exposure (Q1) was used as the reference group

model 1: age, sex, marital, education, residence, smoking status, drinking status

model 2: age, sex, marital, education, residence, smoking status, drinking status, BUN, TC, HDL-C, CRP, UA, PLT, lung disease status, depression status

model 3: age, sex, marital, education, residence, smoking status, drinking status, BUN, TC, HDL-C, CRP, UA, PLT, lung disease status, depression status, solid fuel use for cooking

**Fig. 1:**
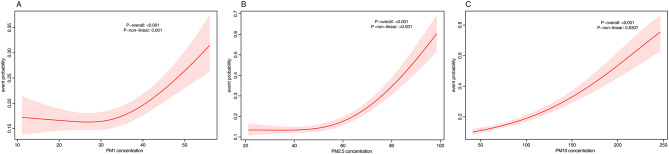
Concentration-response associations of particulate matter (PM_1_, PM_2.5_, and PM_10_) concentrations with event probability. Panels **A**, **B**, and **C** illustrate the concentration-response relationships between particulate matter concentrations (PM_1_, PM_2.5_, and PM_10_) and event probability. The dashed areas represent the 95% confidence intervals (CI). The models are adjusted for relevant confounders. PM_1_ refers to particulate matter with an aerodynamic diameter less than 1 μm, PM_2.5_ to particulate matter less than 2.5 μm, and PM_10_ to particulate matter less than 10 μm. *P*-values for overall effects (*P*-overall) and non-linear relationships (*P*-non-linear) are indicated for each panel

Additionally, the concentration-response (C-R) relationship between air pollutants and event probability is shown in Fig. 1. Analysis using RCS models revealed a significant nonlinear relationship between PM_1_ and PM_2.5_ and event probability (*P* -nonlinear = 0.001 and < 0.001, respectively), while the C-R curve for PM_10_ did not significantly deviate from linearity (*P* -nonlinear = 0.6307). These findings confirm a robust association between long-term air pollution exposure and elevated CVD risk, consistent across models and pollutants.

### Stratified analysis of CKM stages

**Fig. 2:**
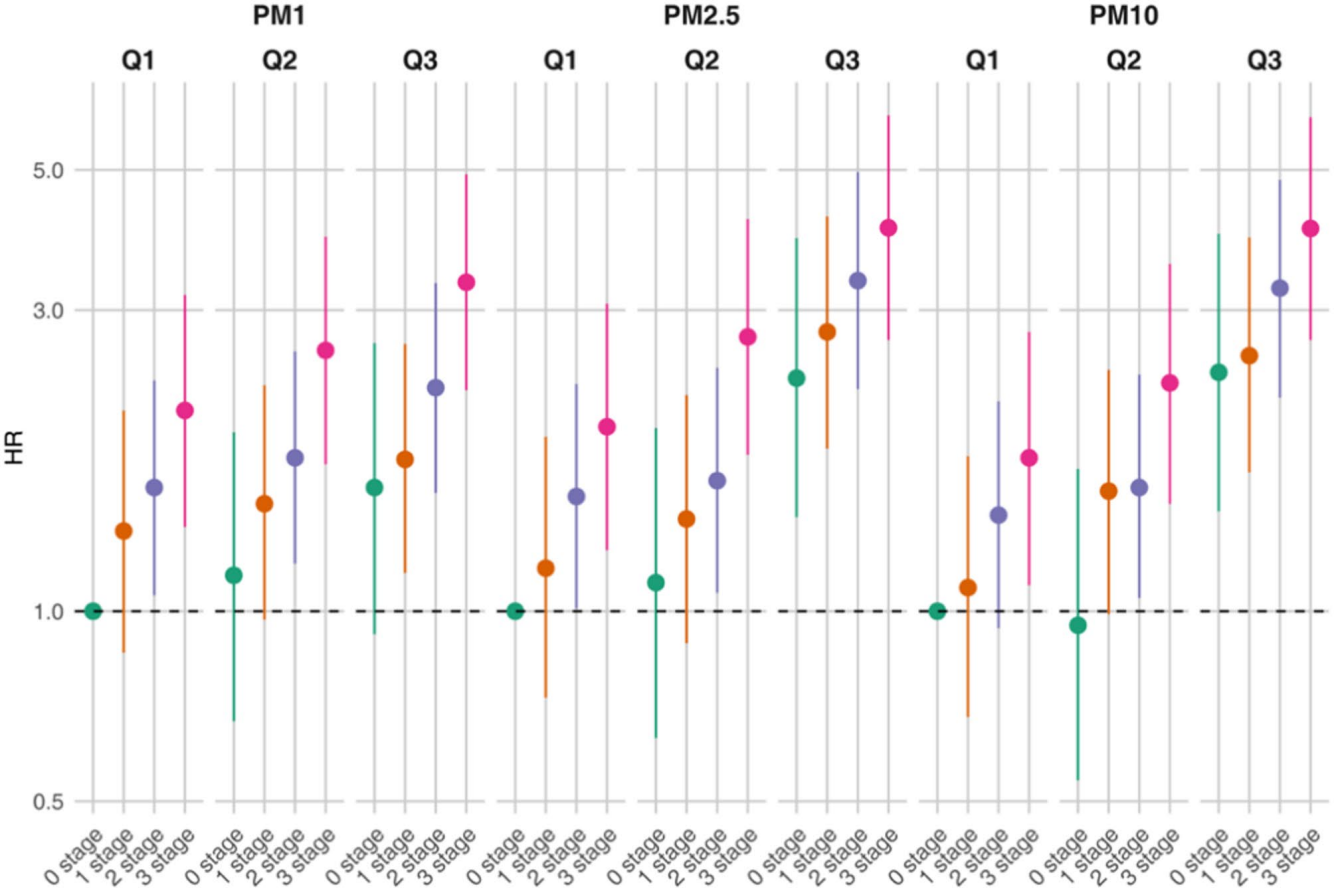
Combined Effects of Air Pollutant Exposure and Disease Stage on Hazard Ratios (HR). This figure illustrates the joint effects of particulate matter concentrations (PM_1_, PM_2.5_, and PM_10_) and disease stages (Stages 0 to 3) on HR. Quartiles of pollutant concentrations (Q1, Q2, Q3) are plotted for each PM size, with a reference line at HR = 1. The HR values represent the risk associated with increasing concentrations of each pollutant across the different stages of the disease. Model adjusted for relevant confounders. CI, confidence interval; PM_1_, particulate matter with an aerodynamic diameter less than 1 μm; PM_2.5_, particulate matter with an aerodynamic diameter less than 2.5 μm; PM_10_, particulate matter with an aerodynamic diameter less than 10 μm

Figure 2 illustrates the combined effects of different CKM stages and air pollution exposure levels on CVD risk (Additional file 1: Table S1). In the fully adjusted model, individuals in the high exposure group (Q3) consistently exhibited a significantly higher risk of CVD compared to those in the medium (Q2) and low (Q1) exposure groups across all pollutants and CKM stages. CVD risk increased progressively across CKM stages, with stage 3 showing the highest risk under all exposure levels, followed by stages 2, 1, and 0. Specifically, under high PM_1_ exposure, the CVD risk was highest in stage 3 (HR = 3.32, 95% CI: 2.24–4.92, *P* < 0.0001), significantly higher than in stage 2 (HR = 2.26, 95% CI: 1.54–3.31, *P* < 0.0001) and stage 1 (HR = 1.74, 95% CI: 1.15–2.65, *P* = 0.01). The CVD risk in stage 0 was the lowest (HR = 1.57, 95% CI: 0.92–2.66, *P* = 0.10), although not statistically significant. This trend was consistent across other pollutants (PM_2.5_ and PM_10_). The graded increase in risk across CKM stages, particularly under high PM1 and PM2.5 exposure, aligns with our hypothesis that individuals at more advanced CKM stages are more vulnerable to pollution-related cardiovascular events.

### PAF for CVD attributable to Long-term exposure to PM_1_, PM_2.5_, and PM_10_

**Fig. 3:**
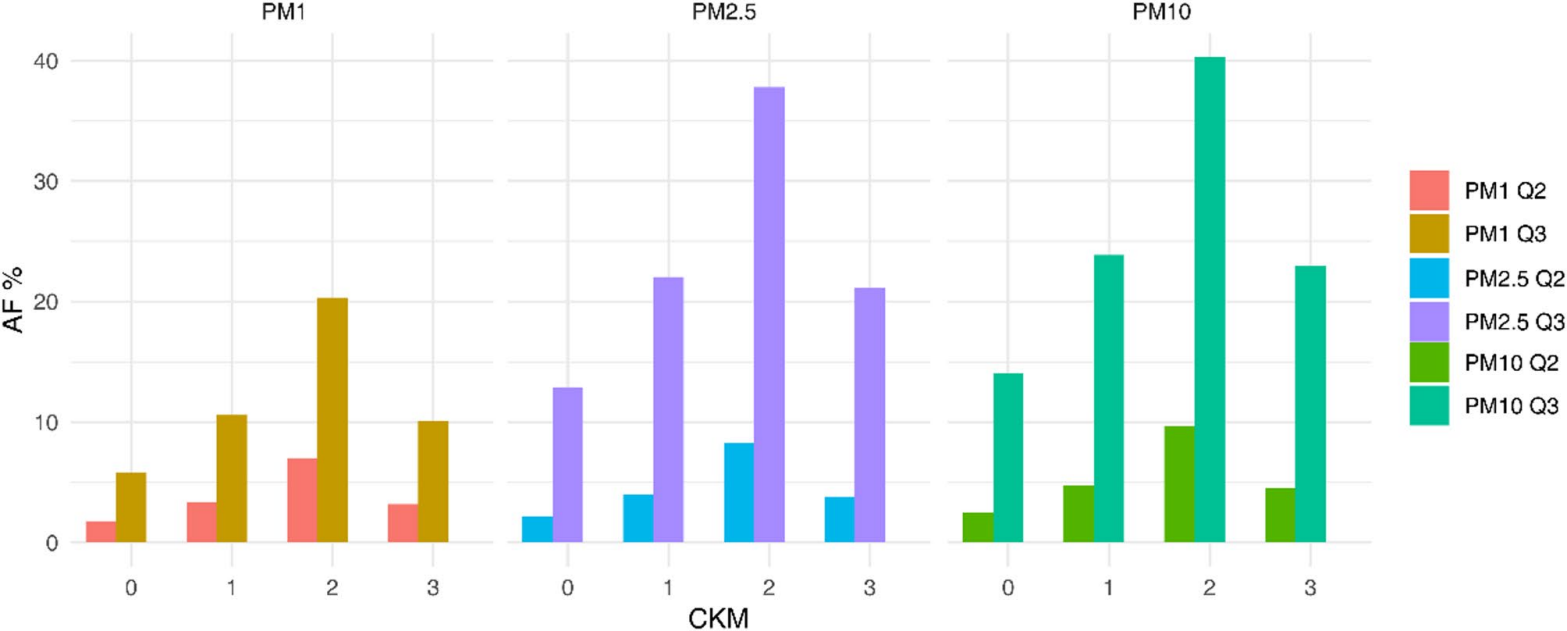
Population Attributable Fraction of CKM Stages by PM_1_, PM_2.5_, and PM_10_ Exposure Levels. This figure displays the attributable fraction (AF%) for different stages of CKM due to exposure to particulate matter concentrations (PM_1_, PM_2.5_, PM_10_) across different exposure quartiles (Q2, Q3). The bars represent the AF% for each CKM stage (Stages 0 to 3), with colors indicating the pollutant concentration quartiles. Quartile designations are as follows: Q2 represents low exposure, and Q3 represents high exposure. Model adjustments include relevant factors such as sex, age, education, residence, and other confounders. PM_1_ refers to particulate matter with an aerodynamic diameter less than 1 μm, PM_2.5_ to particulate matter less than 2.5 μm, and PM_10_ to particulate matter less than 10 μm

Figure 3 shows the PAF for CVD attributable to exposure to PM_1_, PM_2.5_, and PM_10_ at different CKM stages (Additional file 1: Table S2). PAF values varied by pollutant type, exposure level, and CKM stage, indicating notable heterogeneity. For PM_2.5_, the PAF in the medium exposure group (Q2) in stage 1 was 4.02%, increasing to 22.05% in the high exposure group (Q3). In stage 2, the highest PAF was observed for PM_10_ in the high exposure group (Q3), reaching 40.31%. The PAF for PM_2.5_ in stage 2, Q3 was also elevated at 37.84%, substantially higher than in other CKM stages. Although PAFs for PM1 were generally lower than those for other pollutants, they still reached 20.36% in the high-exposure group at CKM stage 2.

### Subgroup analysis

**Fig. 4:**
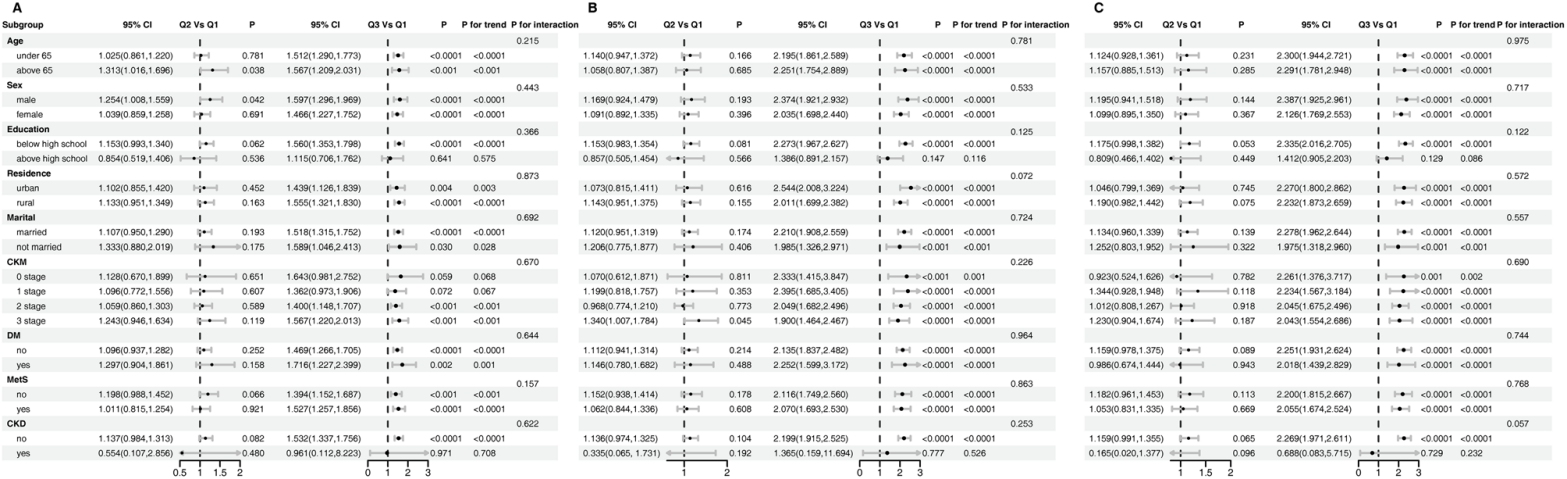
Hazard Ratios (HR) for Different Subgroups by Exposure Quartiles (Q2 vs. Q1, Q3 vs. Q1). Panels **A**, **B**, and **C** display subgroup analyses of hazard ratios (HR) for various factors (age, sex, education, residence, marital status, CKM stage, diabetes, metabolic syndrome, and CKD status) across quartiles of exposure (Q2 vs. Q1, Q3 vs. Q1). The HR values are shown along with 95% confidence intervals (CI), and *P*-values for trend and interaction. A dashed line at HR = 1 indicates no effect, and values greater than 1 suggest an increased risk. The *P*-values for each subgroup comparison indicate the significance of the association. These models are adjusted for relevant confounders

Figure 4 presents the association between air pollution exposure and CVD incidence across different subgroups. No significant interaction was observed across age, sex, education level, and marital status subgroups (*P*-interaction > 0.05). However, within subgroups stratified by CKM stage, the risk of CVD increased significantly with higher levels of air pollutant exposure, consistent with the overall findings. Among participants in CKM stage 2 and stage 3, CVD risk was approximately 1.5 to 2 times higher in the high exposure groups for PM_2.5_ and PM_10_ compared to the low exposure groups. In contrast, the CKD subgroup did not show a statistically significant increase in CVD risk across different air pollution exposure levels.

### Sensitivity analysis

Using 2015 as the baseline and CVD incidence in 2018 as the outcome, we assessed the relationship between air pollution exposure and CVD risk using univariate and multivariate Cox regression models. The overall trends were consistent with the main analysis. However, due to the shorter follow-up period, several associations did not reach statistical significance (Additional file 1: Tables S3–S5).

## Discussion

The concept of CKM introduces a novel understanding of the complex interactions between the cardiovascular, renal, and metabolic systems, framing them as a syndrome that encompasses various pathological features associated with dysfunction and obesity [15, 26]. This framework provides a theoretical foundation for the prevention and evaluation of cardiovascular outcomes from a multidisciplinary perspective [27]. This study provides the first large-scale, longitudinal evidence linking ambient particulate matter exposure to CVD risk across different CKM syndrome stages. By incorporating CKM staging into environmental epidemiology, we move beyond traditional risk stratification and uncover clinically meaningful heterogeneity in susceptibility. While previous studies have established the general link between air pollution and cardiovascular outcomes [4, 22, 28], our findings uniquely demonstrate that this association is significantly magnified in individuals with more advanced CKM stages, thus emphasizing the interaction between environmental and internal metabolic stressors. These results directly address our research hypothesis by showing that CKM stage modifies the air pollution–CVD relationship in a progressive, biologically coherent manner.

We observed a progressive increase in CVD risk across advancing CKM stages, even under equivalent levels of air pollution exposure. This pattern is consistent with prior evidence on the interaction between air pollution and metabolic health deterioration. According to the American Heart Association’s CKM framework, dysfunctional or excessive adiposity is a central driver of vascular dysfunction in CKM syndrome [8]. Supporting this, previous studies have demonstrated a positive association between long-term air pollution exposure and various obesity-related anthropometric indicators, which tend to rise significantly with increasing pollutant concentrations [29]. These effects are likely mediated through multiple biological pathways, including systemic inflammation, oxidative stress, and neuroendocrine disruption. One plausible mechanism is the pollution-induced alteration in insulin sensitivity, which contributes to metabolic dysregulation and increases cardiovascular susceptibility [10]. Insulin resistance and obesity have both been recognized as key factors in predicting CVD risk among CKM populations [30]. In addition, obesity may modulate the impact of air pollution on the development of type 2 diabetes and its cardiovascular complications [10, 31]. Renal dysfunction is another critical predictor of cardiovascular outcomes in patients at CKM stage 2 or above. Numerous studies have shown strong associations between air pollution and the incidence and mortality of CKD [11, 32], as well as the onset of hypertension [9]. In our study, these multiple risk factors may interact synergistically with pollutant exposure to exacerbate CVD risk. Individuals at CKM stage 3, in particular, are likely to exhibit severe insulin resistance, glomerular filtration abnormalities, and cardiac remodeling—each of which heightens vulnerability to pollutant-induced toxicity [33, 34]. Taken together, our findings reinforce the concept that CKM staging functions as a biological amplifier of environmental risk.

The nonlinear concentration–response relationships identified for PM_1_ and PM_2.5_—marked by a steep increase in CVD risk beyond specific exposure thresholds—underscore critical implications for environmental health policy. In 2021, the World Health Organization (WHO) updated its global air quality guidelines to reflect accumulating epidemiological and toxicological evidence indicating that adverse cardiovascular outcomes may occur even at relatively low concentrations of fine particulate matter. Accordingly, the annual recommended limit for PM_2.5_ was revised downward from 10 µg/m^3^ to 5 µg/m^3^. The present findings lend further empirical support to this guideline revision by corroborating the absence of a clearly defined safe exposure threshold for PM_2.5_ in relation to CVD risk [35]. Notably, our exposure–response analyses suggest that such thresholds may not be invariant across populations but are instead modulated by underlying clinical vulnerability, particularly as defined by CKM stage. These results highlight the imperative for future air quality standards—both at the global and regional levels—to incorporate differential susceptibility across subpopulations. These findings have important policy implications, indicating that conventional “one-threshold-for-all” regulatory approaches may inadequately protect cardiometabolically vulnerable populations and should be replaced with precision-based environmental health policies.

Beyond relative risk estimates, our stage-specific analysis of PAFs offers pragmatic insights for prevention. Notably, individuals classified as CKM stage 2 exhibited the highest proportion of CVD burden attributable to air pollution exposure—surpassing that of both earlier and later CKM stages. This is particularly striking given that stage 2 is generally characterized by the emergence of metabolic syndrome and early renal dysfunction, suggesting that it may represent a critical threshold in pathophysiological resilience. Supporting this interpretation, prior studies have found that inflammatory markers differ most significantly between CKM stages 1 and 2, and between stages 2 and 3, whereas differences between stages 0 and 1 or between 3 and 4 are comparatively modest [36]. Inflammation serves as a central initiator and amplifier of multiple CKM components, playing a bidirectional role in promoting metabolic dysregulation, vascular stiffness, and oxidative stress [37, 38]. Air pollution, in turn, has been shown to elevate circulating levels of oxidative stress products and pro-inflammatory mediators, which are implicated in the development of cardiometabolic dysfunction [7]. These mechanisms may partially account for the heightened sensitivity to air pollution observed in stage 2 individuals. According to the AHA framework, CKM stage 2 management emphasizes lifestyle modification and pharmacologic intervention to prevent disease progression [6]. Notably, the relationship between air pollution and CVD is thought to be partly mediated by lifestyle factors, and adherence to healthy behaviors has been shown to significantly attenuate pollution-related cardiovascular risk, especially in individuals exposed to high pollutant levels [28]. From a public health standpoint, these findings underscore the need for targeted health management strategies tailored to CKM stage. Current screening programs often prioritize individuals with overt disease, which may result in missed opportunities for early intervention. As CKM stage 2 represents a phase of heightened vulnerability while retaining modifiability, prioritizing individuals at this stage in both pollution control policies and clinical screening may offer the greatest preventive benefit.

The stratified analysis revealed no significant interactions between sex, age group, education level, residence, marital status, CKM stage, and air pollution, indicating that the effects of CKM stage and air pollution are independent of each other. However, we observed that the risk of CVD in individuals with at least a high school education was not significantly influenced by air pollution, which may be attributed to lifestyle changes and other factors associated with higher education levels [39, 40]. Additionally, we found that CKD patients did not exhibit a significant increase in CVD risk across varying levels of air pollution exposure. This may be because air pollution primarily affects CVD risk through mechanisms such as inflammation and insulin resistance, whereas CKD patients, compared to healthy individuals, are more likely to be on pharmacological treatments. The use of angiotensin receptor blockers (ARBs), angiotensin-converting enzyme inhibitors (ACEIs), and statins in CKD patients may mitigate the effects of air pollution on kidney function decline through antioxidant stress mechanisms [41–43]. However, since the CHARLS dataset does not include information on medication use, further analysis and interpretation of this finding are limited. Future research should focus on examining the metabolic characteristics and distinct risk factors across different CKM stages to facilitate the development of effective preventive measures and behavioral interventions targeting CVD.

This study has several limitations that should be considered. First, due to privacy constraints, air pollution exposure was estimated at the county level rather than by exact residential address, which may reduce exposure specificity. Second, although baseline characteristics differed across exposure groups, we did not implement formal matching. However, we applied stratification and multivariable adjustment, which are standard for estimating population-level associations. Future studies may consider matching or causal inference methods to strengthen exchangeability. Third, several potential confounders—such as medication use, dietary intake, physical activity, and genetic background—were not available in the CHARLS dataset, and thus unmeasured confounding cannot be ruled out. Fourth, CKM stage was assessed at baseline without accounting for longitudinal progression. While our 7-year follow-up allowed for meaningful risk estimation, longer follow-up could better capture cumulative and delayed effects. Despite these limitations, the consistency of findings across models and subgroups supports the robustness of our conclusions and highlights directions for future research to enhance exposure assessment and causal interpretation.

## Conclusion

This study provides robust longitudinal evidence that long-term exposure to PM_1_, PM_2.5_, and PM_10_ is significantly associated with increased risk of CVD, with risk gradients observed across different stages of CKM syndrome. The strongest hazard ratios were found in individuals at CKM stage 3, while the highest PAF was observed at stage 2, indicating that intermediate disease stages may represent a crucial window for preventive intervention. These findings highlight the importance of incorporating CKM staging into cardiovascular risk assessment and environmental health policy, particularly in regions with high levels of ambient air pollution.

Based on these findings, future research should focus on four key directions: (1) improving spatial and temporal resolution in individual-level exposure assessments; (2) tracking dynamic progression of CKM stages over time to understand temporal interactions with pollutant exposure; (3) elucidating biological pathways—such as systemic inflammation and insulin resistance—through integrated biomarker and omics-based approaches; and (4) applying advanced causal inference methods to better isolate the independent effects of pollutants from confounding variables. Such efforts will be essential to developing targeted, stage-specific interventions and informing precision-based environmental health regulations. Overall, our findings underscore the heightened cardiometabolic vulnerability to air pollution and call for integrative strategies that link clinical risk staging with tailored environmental protection policies.

## Electronic supplementary material

Below is the link to the electronic supplementary material.

Supplementary Material 1

## Data Availability

The data can be accessed from the China Health and Retirement Longitudinal Study (CHARLS) (http://charls.pku.edu.cn/) with application.

## Abbreviations

CKM: Cardiovascular-renal-metabolic syndrome
CVD: Cardiovascular disease
CHARLS: China health and retirement longitudinal study
PM_1_: Particulate matter with aerodynamic diameter ≤ 1 μm
PM_2.5_: Particulate matter with aerodynamic diameter ≤ 2.5 μm
PM_10_: Particulate matter with aerodynamic diameter ≤ 10 μm
HR: Hazard ratio
PAF: Population attributable fraction
CI: Confidence interval
AHA: American heart association
CKD: Chronic kidney disease
eGFR: Estimated glomerular filtration rate
C-MDRD: Chinese modification of diet in renal disease
KDIGO: Kidney disease: improving global outcomes
ASCVD: Atherosclerotic cardiovascular disease
TC: Total cholesterol
HDL-C: High-density lipoprotein cholesterol
LDL-C: Low-density lipoprotein cholesterol
TG: Triglycerides
BUN: Blood urea nitrogen
CRP: C-reactive protein
UA: Uric acid
PLT: Platelet count
FBG: Fasting blood glucose
Scr: Serum creatinine
CES-D-10: 10-item center for epidemiologic studies depression scale
RCS: Restricted cubic splines
Q1/Q2/Q3: Quartile 1/Quartile 2/Quartile 3 (exposure levels)
SD: Standard deviation
AF%: Attributable fraction percentage
C-R: Concentration-response
T2DM: Type 2 diabetes mellitus
ARBs: Angiotensin receptor blockers
ACEIs: Angiotensin-converting enzyme inhibitors
CHAP: China high air pollutants
HbA1c: Glycated hemoglobin
IRB: Institutional review board
