# Supplementary material for: Long-term air pollution exposure and cardiovascular disease risk across cardiovascular-renal-metabolic stages: a nationwide study
Source: BMC Public Health. 2025 Jul 2;25:2179. doi: 10.1186/s12889-025-23348-1 (PMC12220752; doi:10.1186/s12889-025-23348-1)
Supplement: Supplementary file 1 — Supplementary Material 1 [file 12889_2025_23348_MOESM1_ESM.docx]

**Supplementary Material**
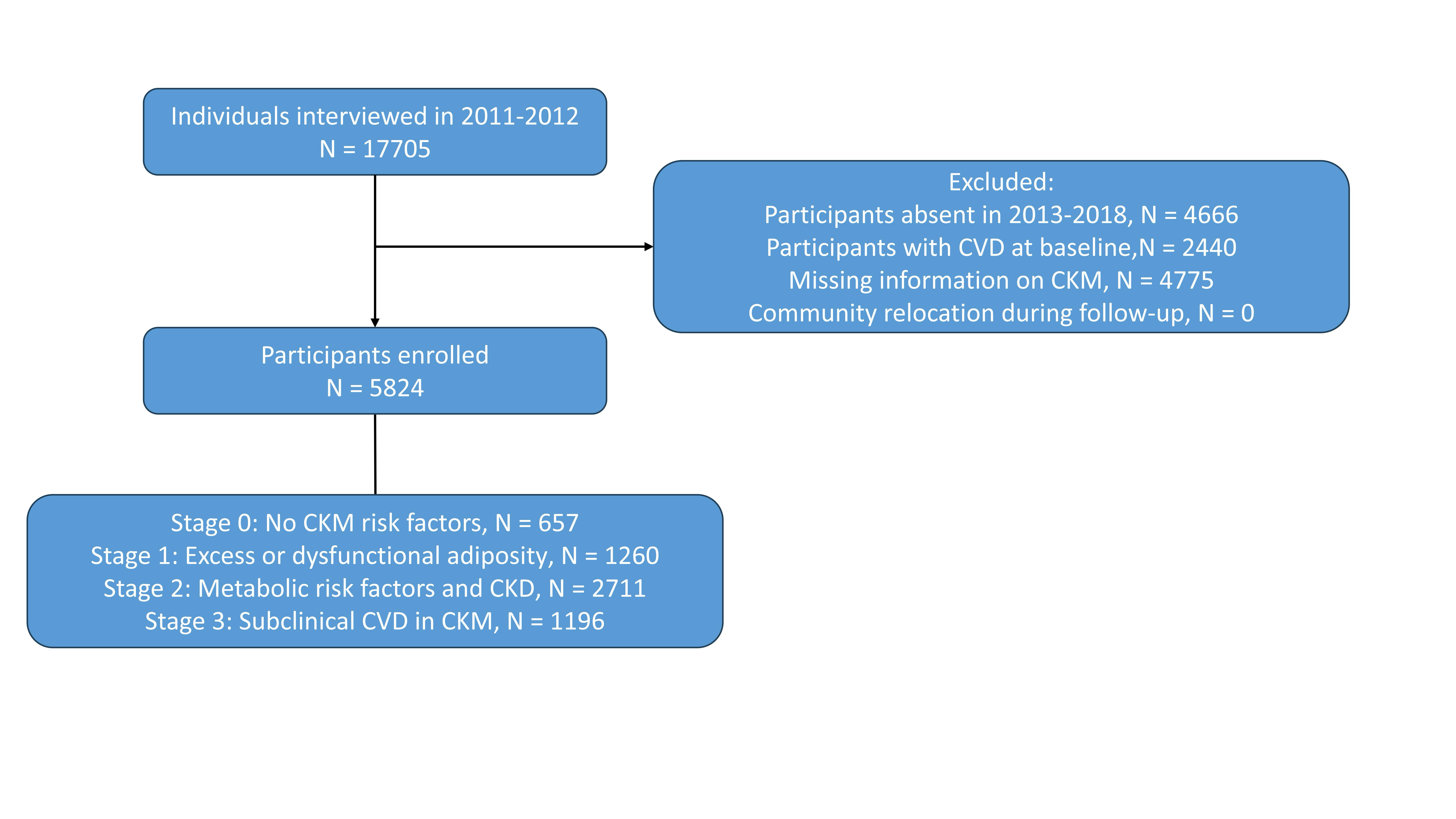


**Figure S1.** Study flowchart

**Table S1. Stratified Analysis Data**

| **character** | | **crude model** | | **Model 1** | | **Model 2** | | **Model 3** | |
| --- | --- | --- | --- | --- | --- | --- | --- | --- | --- |
|  |  | 95%CI | P | 95%CI | P | 95%CI | P | 95%CI | P |
| **PM1** | | | | | | | | | |
| 0 stage | Q1 | ref |  | ref |  | ref |  | ref |  |
|  | Q2 | 1.13(0.67,1.90) | 0.65 | 1.06(0.63,1.79) | 0.82 | 1.14(0.68,1.92) | 0.62 | 1.14(0.67,1.92) | 0.63 |
|  | Q3 | 1.64(0.98,2.76) | 0.06 | 1.63(0.98,2.74) | 0.06 | 1.56(0.92,2.66) | 0.10 | 1.57(0.92,2.66) | 0.10 |
| 1 stage | Q1 | 1.36(0.88,2.10) | 0.17 | 1.33(0.86,2.06) | 0.20 | 1.32(0.85,2.06) | 0.21 | 1.34(0.86,2.08) | 0.19 |
|  | Q2 | 1.49(0.97,2.27) | 0.07 | 1.41(0.92,2.16) | 0.11 | 1.48(0.97,2.28) | 0.07 | 1.48(0.97,2.28) | 0.07 |
|  | Q3 | 1.85(1.22,2.79) | 0.004 | 1.77(1.17,2.68) | 0.01 | 1.74(1.15,2.65) | 0.01 | 1.74(1.15,2.65) | 0.01 |
| 2 stage | Q1 | 1.82(1.24,2.67) | 0.002 | 1.63(1.11,2.40) | 0.01 | 1.57(1.06,2.31) | 0.02 | 1.57(1.06,2.32) | 0.02 |
|  | Q2 | 1.93(1.32,2.82) | <0.001 | 1.72(1.17,2.53) | 0.01 | 1.75(1.19,2.58) | 0.005 | 1.75(1.19,2.58) | 0.005 |
|  | Q3 | 2.55(1.75,3.71) | <0.0001 | 2.33(1.60,3.41) | <0.0001 | 2.26(1.54,3.31) | <0.0001 | 2.26(1.54,3.31) | <0.0001 |
| 3 stage | Q1 | 2.58(1.72,3.87) | <0.0001 | 2.28(1.51,3.44) | <0.0001 | 2.08(1.36,3.17) | <0.001 | 2.08(1.36,3.17) | <0.001 |
|  | Q2 | 3.21(2.16,4.78) | <0.0001 | 2.77(1.85,4.16) | <0.0001 | 2.59(1.71,3.92) | <0.0001 | 2.59(1.71,3.92) | <0.0001 |
|  | Q3 | 4.05(2.76,5.94) | <0.0001 | 3.46(2.35,5.11) | <0.0001 | 3.32(2.24,4.92) | <0.0001 | 3.32(2.24,4.92) | <0.0001 |
| **PM2.5** | | | | | | | | | |
| 0 stage | Q1 | ref |  | ref |  | ref |  | ref |  |
|  | Q2 | 1.07(0.61,1.87) | 0.81 | 1.05(0.60,1.84) | 0.86 | 1.11(0.64,1.95) | 0.71 | 1.11(0.63,1.95) | 0.71 |
|  | Q3 | 2.34(1.42,3.86) | <0.001 | 2.38(1.44,3.93) | <0.001 | 2.34(1.41,3.90) | 0.001 | 2.34(1.41,3.90) | 0.001 |
| 1 stage | Q1 | 1.17(0.73,1.88) | 0.51 | 1.17(0.73,1.88) | 0.52 | 1.16(0.72,1.87) | 0.54 | 1.17(0.73,1.89) | 0.51 |
|  | Q2 | 1.4(0.90,2.19) | 0.13 | 1.37(0.88,2.15) | 0.16 | 1.4(0.89,2.20) | 0.14 | 1.4(0.89,2.20) | 0.14 |
|  | Q3 | 2.8(1.84,4.26) | <0.0001 | 2.75(1.81,4.19) | <0.0001 | 2.77(1.81,4.23) | <0.0001 | 2.77(1.81,4.22) | <0.0001 |
| 2 stage | Q1 | 1.79(1.20,2.68) | 0.005 | 1.63(1.09,2.44) | 0.02 | 1.52(1.01,2.29) | 0.05 | 1.52(1.01,2.29) | 0.04 |
|  | Q2 | 1.73(1.16,2.59) | 0.01 | 1.59(1.06,2.39) | 0.02 | 1.61(1.07,2.43) | 0.02 | 1.61(1.07,2.43) | 0.02 |
|  | Q3 | 3.67(2.49,5.42) | <0.0001 | 3.44(2.33,5.09) | <0.0001 | 3.33(2.24,4.95) | <0.0001 | 3.34(2.25,4.96) | <0.0001 |
| 3 stage | Q1 | 2.57(1.67,3.95) | <0.0001 | 2.19(1.42,3.40) | <0.001 | 1.96(1.26,3.07) | 0.003 | 1.96(1.25,3.07) | 0.003 |
|  | Q2 | 3.45(2.28,5.21) | <0.0001 | 2.98(1.96,4.55) | <0.0001 | 2.72(1.77,4.19) | <0.0001 | 2.72(1.77,4.18) | <0.0001 |
|  | Q3 | 4.89(3.28,7.27) | <0.0001 | 4.16(2.78,6.22) | <0.0001 | 4.05(2.69,6.10) | <0.0001 | 4.05(2.69,6.10) | <0.0001 |
| **PM10** | | | | | | | | | |
| 0 stage | Q1 | ref |  | ref |  | ref |  | ref |  |
|  | Q2 | 0.92(0.52,1.62) | 0.78 | 0.91(0.52,1.61) | 0.75 | 0.95(0.54,1.68) | 0.87 | 0.95(0.54,1.68) | 0.87 |
|  | Q3 | 2.27(1.38,3.73) | 0.001 | 2.4(1.46,3.95) | <0.001 | 2.39(1.44,3.96) | <0.001 | 2.39(1.44,3.96) | <0.001 |
| 1 stage | Q1 | 1.12(0.70,1.79) | 0.64 | 1.12(0.70,1.79) | 0.65 | 1.09(0.67,1.75) | 0.74 | 1.09(0.68,1.76) | 0.72 |
|  | Q2 | 1.5(0.97,2.33) | 0.07 | 1.5(0.97,2.32) | 0.07 | 1.54(0.99,2.40) | 0.06 | 1.55(0.99,2.41) | 0.05 |
|  | Q3 | 2.5(1.63,3.81) | <0.0001 | 2.52(1.64,3.85) | <0.0001 | 2.54(1.65,3.91) | <0.0001 | 2.54(1.66,3.91) | <0.0001 |
| 2 stage | Q1 | 1.68(1.12,2.51) | 0.01 | 1.54(1.03,2.31) | 0.04 | 1.42(0.94,2.14) | 0.09 | 1.42(0.94,2.15) | 0.09 |
|  | Q2 | 1.7(1.13,2.54) | 0.01 | 1.58(1.05,2.36) | 0.03 | 1.57(1.04,2.37) | 0.03 | 1.57(1.05,2.37) | 0.03 |
|  | Q3 | 3.43(2.32,5.06) | <0.0001 | 3.26(2.21,4.83) | <0.0001 | 3.24(2.18,4.81) | <0.0001 | 3.25(2.18,4.82) | <0.0001 |
| 3 stage | Q1 | 2.36(1.52,3.66) | <0.001 | 1.95(1.25,3.06) | 0.004 | 1.74(1.10,2.76) | 0.02 | 1.75(1.10,2.77) | 0.02 |
|  | Q2 | 2.9(1.91,4.41) | <0.0001 | 2.52(1.64,3.85) | <0.0001 | 2.29(1.48,3.54) | <0.001 | 2.3(1.48,3.55) | <0.001 |
|  | Q3 | 4.82(3.25,7.15) | <0.0001 | 4.09(2.74,6.10) | <0.0001 | 4.04(2.69,6.06) | <0.0001 | 4.04(2.69,6.06) | <0.0001 |

model 1: age, sex, marital, education, residence, smoking status, drinking status.

model 2: age, sex, marital, education, residence, smoking status, drinking status, BUN, TC, HDL-C, CRP, UA, PLT, lung disease status, depression status.

model 3: age, sex, marital, education, residence, smoking status, drinking status, BUN, TC, HDL-C, CRP, UA, PLT, lung disease status, depression status, solid fuel use for cooking.

**Table S2. Population Attributable Fraction Data**

| CKM stage | Level | HR | Proportion | PAF |
| --- | --- | --- | --- | --- |
| PM1 | | | | |
| 0 stage | Q1 | 1 | 0.1128091 | 0 |
|  | Q2 | 1.161607 | 0.1128091 | 0.01790428 |
|  | Q3 | 1.549301 | 0.1128091 | 0.05835034 |
| 1 stage | Q1 | 1 | 0.2163462 | 0 |
|  | Q2 | 1.161607 | 0.2163462 | 0.03378185 |
|  | Q3 | 1.549301 | 0.2163462 | 0.1062164 |
| 2 stage | Q1 | 1 | 0.4654876 | 0 |
|  | Q2 | 1.161607 | 0.4654876 | 0.06996286 |
|  | Q3 | 1.549301 | 0.4654876 | 0.20362674 |
| 3 stage | Q1 | 1 | 0.2053571 | 0 |
|  | Q2 | 1.161607 | 0.2053571 | 0.03212107 |
|  | Q3 | 1.549301 | 0.2053571 | 0.10136817 |
| PM2.5 | | | | |
| 0 stage | Q1 | 1 | 0.1128091 | 0 |
|  | Q2 | 1.193676 | 0.1128091 | 0.0213813 |
|  | Q3 | 2.307612 | 0.1128091 | 0.12854823 |
| 1 stage | Q1 | 1 | 0.2163462 | 0 |
|  | Q2 | 1.193676 | 0.2163462 | 0.04021604 |
|  | Q3 | 2.307612 | 0.2163462 | 0.22051403 |
| 2 stage | Q1 | 1 | 0.4654876 | 0 |
|  | Q2 | 1.193676 | 0.4654876 | 0.08269836 |
|  | Q3 | 2.307612 | 0.4654876 | 0.37837117 |
| 3 stage | Q1 | 1 | 0.2053571 | 0 |
|  | Q2 | 1.193676 | 0.2053571 | 0.03825145 |
|  | Q3 | 2.307612 | 0.2053571 | 0.21168433 |
| PM10 | | | | |
| 0 stage | Q1 | 1 | 0.1128091 | 0 |
|  | Q2 | 1.229894 | 0.1128091 | 0.02527851 |
|  | Q3 | 2.450708 | 0.1128091 | 0.14063728 |
| 1 stage | Q1 | 1 | 0.2163462 | 0 |
|  | Q2 | 1.229894 | 0.2163462 | 0.04738007 |
|  | Q3 | 2.450708 | 0.2163462 | 0.23888104 |
| 2 stage | Q1 | 1 | 0.4654876 | 0 |
|  | Q2 | 1.229894 | 0.4654876 | 0.09666794 |
|  | Q3 | 2.450708 | 0.4654876 | 0.4030872 |
| 3 stage | Q1 | 1 | 0.2053571 | 0 |
|  | Q2 | 1.229894 | 0.2053571 | 0.04508196 |
|  | Q3 | 2.450708 | 0.2053571 | 0.22953246 |

**Table S3. Sensitivity Analysis - Association between air pollutant and CVD risk**

|  | PM1 | | | | PM2.5 | | | | PM10 | | | |
| --- | --- | --- | --- | --- | --- | --- | --- | --- | --- | --- | --- | --- |
|  | Q2 | P | Q3 | P | Q2 | P | Q3 | P | Q2 | P | Q3 | P |
| crude model | 1.15(0.97,1.36) | 0.10 | 1.43(1.22,1.67) | <0.0001 | 1.13(0.96,1.34) | 0.15 | 1.46(1.25,1.72) | <0.0001 | 1.11(0.94,1.32) | 0.21 | 1.48(1.26,1.74) | <0.0001 |
| Model 1 | 1.14(0.95,1.36) | 0.15 | 1.46(1.23,1.72) | <0.0001 | 1.1(0.92,1.31) | 0.29 | 1.53(1.29,1.80) | <0.0001 | 1.1(0.92,1.32) | 0.27 | 1.55(1.31,1.83) | <0.0001 |
| Model 2 | 1.17(0.97,1.41) | 0.10 | 1.53(1.29,1.82) | <0.0001 | 1.11(0.93,1.34) | 0.26 | 1.58(1.33,1.89) | <0.0001 | 1.15(0.95,1.38) | 0.15 | 1.64(1.37,1.95) | <0.0001 |
| Model 3 | 1.17(0.97,1.41) | 0.09 | 1.53(1.29,1.82) | <0.0001 | 1.11(0.92,1.33) | 0.28 | 1.58(1.33,1.88) | <0.0001 | 1.14(0.95,1.38) | 0.16 | 1.63(1.36,1.94) | <0.0001 |

Low exposure (Q1) was used as the reference group.

model 1: age, sex, marital, education, residence, smoking status, drinking status.

model 2: age, sex, marital, education, residence, smoking status, drinking status, BUN, TC, HDL-C, CRP, UA, PLT, lung disease status, depression status.

model 3: age, sex, marital, education, residence, smoking status, drinking status, BUN, TC, HDL-C, CRP, UA, PLT, lung disease status, depression status, solid fuel use for cooking.

**Table S4. Sensitivity Analysis - Stratified Analysis Data**

| **character** | | **crude model** | | **Model 1** | | **Model 2** | | **Model 3** | |
| --- | --- | --- | --- | --- | --- | --- | --- | --- | --- |
|  |  | 95%CI | P | 95%CI | P | 95%CI | P | 95%CI | P |
| **PM1** | | | | | | | | | |
| 0 stage | Q1 | ref |  | ref |  | ref |  | ref |  |
|  | Q2 | 1.13(0.67,1.90) | 0.65 | 1.06(0.63,1.79) | 0.82 | 1.14(0.68,1.92) | 0.62 | 1.14(0.67,1.92) | 0.63 |
|  | Q3 | 1.64(0.98,2.76) | 0.06 | 1.63(0.98,2.74) | 0.06 | 1.56(0.92,2.66) | 0.10 | 1.57(0.92,2.66) | 0.10 |
| 1 stage | Q1 | 1.36(0.88,2.10) | 0.17 | 1.33(0.86,2.06) | 0.20 | 1.32(0.85,2.06) | 0.21 | 1.34(0.86,2.08) | 0.19 |
|  | Q2 | 1.49(0.97,2.27) | 0.07 | 1.41(0.92,2.16) | 0.11 | 1.48(0.97,2.28) | 0.07 | 1.48(0.97,2.28) | 0.07 |
|  | Q3 | 1.85(1.22,2.79) | 0.004 | 1.77(1.17,2.68) | 0.01 | 1.74(1.15,2.65) | 0.01 | 1.74(1.15,2.65) | 0.01 |
| 2 stage | Q1 | 1.82(1.24,2.67) | 0.002 | 1.63(1.11,2.40) | 0.01 | 1.57(1.06,2.31) | 0.02 | 1.57(1.06,2.32) | 0.02 |
|  | Q2 | 1.93(1.32,2.82) | <0.001 | 1.72(1.17,2.53) | 0.01 | 1.75(1.19,2.58) | 0.005 | 1.75(1.19,2.58) | 0.005 |
|  | Q3 | 2.55(1.75,3.71) | <0.0001 | 2.33(1.60,3.41) | <0.0001 | 2.26(1.54,3.31) | <0.0001 | 2.26(1.54,3.31) | <0.0001 |
| 3 stage | Q1 | 2.58(1.72,3.87) | <0.0001 | 2.28(1.51,3.44) | <0.0001 | 2.08(1.36,3.17) | <0.001 | 2.08(1.36,3.17) | <0.001 |
|  | Q2 | 3.21(2.16,4.78) | <0.0001 | 2.77(1.85,4.16) | <0.0001 | 2.59(1.71,3.92) | <0.0001 | 2.59(1.71,3.92) | <0.0001 |
|  | Q3 | 4.05(2.76,5.94) | <0.0001 | 3.46(2.35,5.11) | <0.0001 | 3.32(2.24,4.92) | <0.0001 | 3.32(2.24,4.92) | <0.0001 |
| **PM2.5** | | | | | | | | | |
| 0 stage | Q1 | ref |  | ref |  | ref |  | ref |  |
|  | Q2 | 0.92(0.52,1.62) | 0.78 | 0.91(0.52,1.61) | 0.75 | 0.95(0.54,1.68) | 0.87 | 0.95(0.54,1.68) | 0.87 |
|  | Q3 | 2.27(1.38,3.73) | 0.001 | 2.4(1.46,3.95) | <0.001 | 2.39(1.44,3.96) | <0.001 | 2.39(1.44,3.96) | <0.001 |
| 1 stage | Q1 | 1.12(0.70,1.79) | 0.64 | 1.12(0.70,1.79) | 0.65 | 1.09(0.67,1.75) | 0.74 | 1.09(0.68,1.76) | 0.72 |
|  | Q2 | 1.5(0.97,2.33) | 0.07 | 1.5(0.97,2.32) | 0.07 | 1.54(0.99,2.40) | 0.06 | 1.55(0.99,2.41) | 0.05 |
|  | Q3 | 2.5(1.63,3.81) | <0.0001 | 2.52(1.64,3.85) | <0.0001 | 2.54(1.65,3.91) | <0.0001 | 2.54(1.66,3.91) | <0.0001 |
| 2 stage | Q1 | 1.68(1.12,2.51) | 0.01 | 1.54(1.03,2.31) | 0.04 | 1.42(0.94,2.14) | 0.09 | 1.42(0.94,2.15) | 0.09 |
|  | Q2 | 1.7(1.13,2.54) | 0.01 | 1.58(1.05,2.36) | 0.03 | 1.57(1.04,2.37) | 0.03 | 1.57(1.05,2.37) | 0.03 |
|  | Q3 | 3.43(2.32,5.06) | <0.0001 | 3.26(2.21,4.83) | <0.0001 | 3.24(2.18,4.81) | <0.0001 | 3.25(2.18,4.82) | <0.0001 |
| 3 stage | Q1 | 2.36(1.52,3.66) | <0.001 | 1.95(1.25,3.06) | 0.004 | 1.74(1.10,2.76) | 0.02 | 1.75(1.10,2.77) | 0.02 |
|  | Q2 | 2.9(1.91,4.41) | <0.0001 | 2.52(1.64,3.85) | <0.0001 | 2.29(1.48,3.54) | <0.001 | 2.3(1.48,3.55) | <0.001 |
|  | Q3 | 4.82(3.25,7.15) | <0.0001 | 4.09(2.74,6.10) | <0.0001 | 4.04(2.69,6.06) | <0.0001 | 4.04(2.69,6.06) | <0.0001 |
| **PM10** | | | | | | | | | |
| 0 stage | Q1 | ref |  | ref |  | ref |  | ref |  |
|  | Q2 | 1.07(0.61,1.87) | 0.81 | 1.05(0.60,1.84) | 0.86 | 1.11(0.64,1.95) | 0.71 | 1.11(0.63,1.95) | 0.71 |
|  | Q3 | 2.34(1.42,3.86) | <0.001 | 2.38(1.44,3.93) | <0.001 | 2.34(1.41,3.90) | 0.001 | 2.34(1.41,3.90) | 0.001 |
| 1 stage | Q1 | 1.17(0.73,1.88) | 0.51 | 1.17(0.73,1.88) | 0.52 | 1.16(0.72,1.87) | 0.54 | 1.17(0.73,1.89) | 0.51 |
|  | Q2 | 1.4(0.90,2.19) | 0.13 | 1.37(0.88,2.15) | 0.16 | 1.4(0.89,2.20) | 0.14 | 1.4(0.89,2.20) | 0.14 |
|  | Q3 | 2.8(1.84,4.26) | <0.0001 | 2.75(1.81,4.19) | <0.0001 | 2.77(1.81,4.23) | <0.0001 | 2.77(1.81,4.22) | <0.0001 |
| 2 stage | Q1 | 1.79(1.20,2.68) | 0.005 | 1.63(1.09,2.44) | 0.02 | 1.52(1.01,2.29) | 0.05 | 1.52(1.01,2.29) | 0.04 |
|  | Q2 | 1.73(1.16,2.59) | 0.01 | 1.59(1.06,2.39) | 0.02 | 1.61(1.07,2.43) | 0.02 | 1.61(1.07,2.43) | 0.02 |
|  | Q3 | 3.67(2.49,5.42) | <0.0001 | 3.44(2.33,5.09) | <0.0001 | 3.33(2.24,4.95) | <0.0001 | 3.34(2.25,4.96) | <0.0001 |
| 3 stage | Q1 | 2.57(1.67,3.95) | <0.0001 | 2.19(1.42,3.40) | <0.001 | 1.96(1.26,3.07) | 0.003 | 1.96(1.25,3.07) | 0.003 |
|  | Q2 | 3.45(2.28,5.21) | <0.0001 | 2.98(1.96,4.55) | <0.0001 | 2.72(1.77,4.19) | <0.0001 | 2.72(1.77,4.18) | <0.0001 |
|  | Q3 | 4.89(3.28,7.27) | <0.0001 | 4.16(2.78,6.22) | <0.0001 | 4.05(2.69,6.10) | <0.0001 | 4.05(2.69,6.10) | <0.0001 |

model 1: age, sex, marital, education, residence, smoking status, drinking status.

model 2: age, sex, marital, education, residence, smoking status, drinking status, BUN, TC, HDL-C, CRP, UA, PLT, lung disease status, depression status.

model 3: age, sex, marital, education, residence, smoking status, drinking status, BUN, TC, HDL-C, CRP, UA, PLT, lung disease status, depression status, solid fuel use for cooking.

**Table S5. Sensitivity Analysis - Subgroup Analysis**

| character | Q2 | p | Q3 | p | p for interaction |
| --- | --- | --- | --- | --- | --- |
| PM1 | | | | | |
| age | | | | | 0.481 |
| above 65 | 1.291(0.986,1.692) | 0.064 | 1.585(1.220,2.059) | <0.001 |  |
| under 65 | 1.056(0.852,1.309) | 0.618 | 1.346(1.102,1.645) | 0.004 |  |
| sex | | | | | 0.794 |
| male | 1.204(0.936,1.549) | 0.149 | 1.406(1.103,1.793) | 0.006 |  |
| female | 1.110(0.886,1.389) | 0.365 | 1.438(1.166,1.775) | <0.001 |  |
| education | | | | | 0.324 |
| below high school | 1.190(0.991,1.428) | 0.062 | 1.496(1.257,1.779) | <0.0001 |  |
| above high school | 0.733(0.395,1.363) | 0.327 | 1.050(0.596,1.849) | 0.866 |  |
| residence | | | | | 0.335 |
| urban | 0.972(0.730,1.295) | 0.846 | 1.294(0.982,1.705) | 0.067 |  |
| rural | 1.270(1.032,1.563) | 0.024 | 1.496(1.232,1.818) | <0.0001 |  |
| marital | | | | | 0.095 |
| married | 1.135(0.949,1.358) | 0.165 | 1.343(1.131,1.594) | <0.001 |  |
| notmarried | 1.274(0.792,2.050) | 0.318 | 2.154(1.403,3.307) | <0.001 |  |
| CKM stage | | | | | 0.553 |
| 0 stage | 1.090(0.532,2.233) | 0.814 | 0.770(0.311,1.907) | 0.572 |  |
| 1 stage | 1.245(0.799,1.941) | 0.333 | 1.462(0.967,2.211) | 0.072 |  |
| 2 stage | 1.087(0.853,1.385) | 0.500 | 1.465(1.160,1.849) | 0.001 |  |
| 3 stage | 1.179(0.878,1.583) | 0.274 | 1.194(0.910,1.566) | 0.201 |  |
| DM | | | | | 0.458 |
| no | 1.212(1.000,1.469) | 0.051 | 1.446(1.202,1.740) | <0.0001 |  |
| yes | 0.944(0.671,1.328) | 0.743 | 1.250(0.916,1.704) | 0.159 |  |
| MetS | | | | | 0.709 |
| no | 1.058(0.847,1.323) | 0.619 | 1.313(1.061,1.625) | 0.012 |  |
| yes | 1.217(0.942,1.572) | 0.133 | 1.454(1.141,1.853) | 0.002 |  |
| CKD | | | | | 0.603 |
| no | 1.122(0.945,1.331) | 0.188 | 1.434(1.223,1.682) | <0.0001 |  |
| yes | 1.121(0.336,3.746) | 0.853 | 0.587(0.061,5.648) | 0.645 |  |
| PM2.5 | | | | | |
| age | | | | | 0.858 |
| above 65 | 1.154(0.885,1.505) | 0.289 | 1.573(1.213,2.038) | <0.001 |  |
| under 65 | 1.101(0.888,1.366) | 0.380 | 1.433(1.171,1.755) | <0.001 |  |
| sex | | | | | 0.572 |
| male | 1.212(0.944,1.557) | 0.132 | 1.430(1.120,1.827) | 0.004 |  |
| female | 1.071(0.855,1.341) | 0.550 | 1.487(1.204,1.835) | <0.001 |  |
| education | | | | | 0.29 |
| below high school | 1.126(0.937,1.352) | 0.206 | 1.572(1.322,1.869) | <0.0001 |  |
| above high school | 0.974(0.530,1.788) | 0.932 | 0.990(0.553,1.772) | 0.973 |  |
| residence | | | | | 0.137 |
| urban | 0.934(0.706,1.236) | 0.631 | 1.449(1.111,1.892) | 0.006 |  |
| rural | 1.266(1.028,1.560) | 0.027 | 1.476(1.209,1.802) | <0.001 |  |
| marital | | | | | 0.02 |
| married | 1.116(0.934,1.334) | 0.228 | 1.356(1.142,1.610) | <0.001 |  |
| not married | 1.259(0.776,2.041) | 0.351 | 2.475(1.602,3.823) | <0.0001 |  |
| CKM stage | | | | | 0.48 |
| 0 stage | 0.941(0.459,1.928) | 0.868 | 0.728(0.299,1.769) | 0.483 |  |
| 1 stage | 1.267(0.824,1.947) | 0.280 | 1.276(0.835,1.949) | 0.260 |  |
| 2 stage | 1.118(0.877,1.424) | 0.367 | 1.570(1.243,1.983) | <0.001 |  |
| 3 stage | 1.037(0.770,1.397) | 0.809 | 1.227(0.933,1.613) | 0.143 |  |
| DM | | | | | 0.905 |
| no | 1.123(0.928,1.359) | 0.234 | 1.407(1.170,1.692) | <0.001 |  |
| yes | 1.139(0.805,1.611) | 0.463 | 1.518(1.101,2.093) | 0.011 |  |
| MetS | | | | | 0.269 |
| no | 1.041(0.838,1.295) | 0.715 | 1.240(1.000,1.539) | 0.050 |  |
| yes | 1.234(0.948,1.606) | 0.117 | 1.623(1.270,2.072) | <0.001 |  |
| CKD | | | | | 0.439 |
| no | 1.127(0.950,1.338) | 0.169 | 1.486(1.265,1.745) | <0.0001 |  |
| yes | 0.600(0.239,1.502) | 0.275 | 0.697(0.084,5.791) | 0.738 |  |
| PM10 | | | | | |
| age | | | | | 0.626 |
| above 65 | 1.220(0.935,1.591) | 0.144 | 1.657(1.281,2.145) | <0.001 |  |
| under 65 | 1.056(0.850,1.312) | 0.623 | 1.429(1.168,1.750) | <0.001 |  |
| sex | | | | | 0.748 |
| male | 1.046(0.814,1.346) | 0.724 | 1.390(1.093,1.767) | 0.007 |  |
| female | 1.175(0.937,1.473) | 0.163 | 1.553(1.255,1.922) | <0.0001 |  |
| education | | | | | 0.206 |
| below high school | 1.128(0.938,1.356) | 0.202 | 1.594(1.341,1.896) | <0.0001 |  |
| above high school | 0.848(0.463,1.552) | 0.592 | 0.928(0.527,1.634) | 0.796 |  |
| residence | | | | | 0.343 |
| urban | 0.960(0.725,1.270) | 0.775 | 1.455(1.118,1.893) | 0.005 |  |
| rural | 1.213(0.982,1.498) | 0.073 | 1.499(1.227,1.831) | <0.0001 |  |
| marital | | | | | 0.034 |
| married | 1.090(0.911,1.304) | 0.348 | 1.372(1.156,1.629) | <0.001 |  |
| not married | 1.319(0.811,2.146) | 0.264 | 2.451(1.596,3.765) | <0.0001 |  |
| CKM stage | | | | | 0.766 |
| 0 stage | 0.828(0.398,1.721) | 0.613 | 0.910(0.389,2.126) | 0.827 |  |
| 1 stage | 1.256(0.812,1.942) | 0.307 | 1.391(0.915,2.113) | 0.122 |  |
| 2 stage | 1.110(0.871,1.414) | 0.399 | 1.516(1.201,1.912) | <0.001 |  |
| 3 stage | 0.968(0.717,1.309) | 0.835 | 1.197(0.908,1.578) | 0.202 |  |
| DM | | | | | 0.611 |
| no | 1.077(0.889,1.304) | 0.451 | 1.390(1.157,1.669) | <0.001 |  |
| yes | 1.243(0.874,1.768) | 0.226 | 1.674(1.209,2.317) | 0.002 |  |
| MetS | | | | | 0.79 |
| no | 1.077(0.865,1.342) | 0.508 | 1.335(1.076,1.655) | 0.009 |  |
| yes | 1.110(0.854,1.443) | 0.437 | 1.484(1.165,1.890) | 0.001 |  |
| CKD | | | | | 0.018 |
| no | 1.140(0.960,1.354) | 0.136 | 1.525(1.298,1.792) | <0.0001 |  |
| yes | 0.332(0.149,0.739) | 0.007 | 0.400(0.051,3.125) | 0.382 |  |

Low exposure (Q1) was used as the reference group.
